# Post COVID-19 Chronic Parenchymal Lung Changes

**DOI:** 10.7759/cureus.25197

**Published:** 2022-05-21

**Authors:** Abdelfattah Touman, Mohammed Kahyat, Adeeb Bulkhi, Mutaz Khairo, Wael Alyamani, Ahmad M Aldobyany, Nabil Ghaleb, Hadeel Ashi, Mohammed Alsobaie, Eid Alqurashi

**Affiliations:** 1 Department of Pulmonology, King Abdullah Medical City, Makkah, SAU; 2 Department of Internal Medicine, Umm Al-Qura University, Makkah, SAU; 3 Department of Radiology, King Abdullah Medical City, Makkah, SAU

**Keywords:** post-covid-19 fibrosis, complication, long covid, interstitial lung disease, covid-19

## Abstract

Introduction: Persistent parenchymal lung changes are an important long-term sequela of COVID-19. There are limited data on this COVID-19 infection sequela characteristics and trajectories. This study aims to evaluate persistent COVID-19-related parenchymal lung changes 10 weeks after acute viral pneumonia and to identify associated risk factors.

Methods: This is a retrospective case-control observational study involving 38 COVID-19 confirmed cases using nasopharyngeal swab reverse transcriptase-polymerase chain reaction (RT-PCR) at King Abdullah Medical City (KAMC) Hospital, Makkah. Patients were recruited from the post-COVID-19 interstitial lung disease (ILD) clinic. Referral to this clinic was based on the pulmonology consultant’s assessment of hospitalized patients suspected of developing COVID-19-related ILD changes during hospitalization.

Results: Thirty-eight patients with parenchymal lung changes were evaluated at the ILD clinic. Nineteen patients who had persistent parenchymal changes 10 weeks after the acute illness (group 1) were compared with 19 control patients who had accelerated clinical and/or radiological improvement (group 2). Group 1 was found to have the more severe clinical and radiological disease, with a higher peak value of inflammatory biomarkers. Two risk factors were identified, neutrophil-lymphocyte ratio (NLR) > 3.13 at admission increases the odds ratio (OR) of chronic parenchymal changes by 6.42 and 5.92 in the univariate and multivariate analyses, respectively. Invasive mechanical ventilation had a more profound effect with ORs of 13.09 and 44.5 in the univariate and multivariate analyses, respectively.

Conclusion: Herein, we found that only receiving invasive mechanical ventilation and having NLR >3.13 at admission were strong risk factors for persistent parenchymal lung changes. Neither the clinical severity of the acute illness nor the radiological one is found to predict this outcome. None of the medications received during the acute illness were found to alter the risk for this post-COVID-19 infection sequelae.

## Introduction

By May 2021, more than 150 million confirmed Coronavirus disease (COVID-19) cases worldwide, and around 3 million deaths were reported [1]. COVID-19 infection has been the focus of numerous studies, nevertheless, the long-term sequelae of COVID-19 infection including the persistent parenchymal lung changes are not well understood. Early reports described persistent symptoms after acute COVID-19 infection [2,3]. These symptoms and organ dysfunction were not limited to the lungs but included psychological, cardiovascular, neurological, hematological, and other system disorders [3,4]. Several observational studies described the persistent parenchymal lung changes, in particular [5-7]. Nevertheless, more studies are needed to fully understand this post-COVID-19 infection sequela. Our study aimed to elaborate on factors that contribute to the development of post-acute COVID-19 pneumonia parenchymal lung changes.

This research has been published in Article: Hongjie Yu, Zhiyuan Chen, Andrew Azman, et al.). Global Landscape of SARS-CoV-2 Genomic Surveillance, Public Availability Extent of Genomic Data, and Epidemic Shaped by Variants; 2021.

## Materials and methods

Ethical approval

The Institutional Review Board of King Abdallah Medical City (KAMC) approved this study with the number (21-763).

Study design and patient selection

This retrospective case-control observational study involved COVID-19 confirmed cases using nasopharyngeal swab by reverse transcription-polymerase chain reaction (RT-PCR) at KAMC, Makkah-Saudi Arabia. Patients were recruited from the post-COVID-19 interstitial lung disease (ILD) clinic. Referral to this clinic was based on a pulmonologist’s assessment of hospitalized patients suspected of developing COVID-19-related parenchymal changes during hospitalization.

Thirty-eight patients were evaluated at the post-COVID-19 ILD clinic, and detailed medical history and physical examination were performed. Laboratory and physiological assessments were requested for all patients; however, follow-up chest computed tomography (CT) was requested for patients with significant residual disease, such as patients with severe symptoms who still required oxygen at home to maintain oxygen saturation for more than two months after discharge and those in whom the prednisolone dose could not be tapered down. These patients were distributed into two groups, patients who had persistent parenchymal lung changes at a follow-up chest CT done at least 10 weeks after the first positive RT-PCR swab “Group 1..” Group 2 “the control group” included patients with earlier clinical and/or radiological improvement.

Inclusion criteria included all adults (age >12 years) and RT-PCR confirmed COVID-19 infection with radiological evidence of pneumonia. We excluded patients diagnosed with fibrotic or other structural lung diseases prior to having COVID-19 infection. Patients who were lost to follow-up after discharge or judged to need a follow-up chest CT and missed their radiology appointments were also excluded. Baseline patient characteristics, including age, sex, smoking history, obesity, comorbidities, and other clinical variables related to acute COVID-19 disease, such as inflammatory markers, treatment intervention, and intensive care unit (ICU) admission, were collected from electronic the patients’ records.

Chest CT image protocol and interpretation

CT scans were acquired without cardiac gating on a 64 slice multidetector CT (SIEMENS SOMATOM Sensation 64) with a 64×0.625 mm collimation and spiral pitch factor of 1.3. Scans were obtained in the craniocaudal direction, supine position and during end-inspiration without an intravenous contrast agent with a standard dose scanning protocol. For those patients who had a clinical suspicion of pulmonary embolism (PE), an additional CT pulmonary angiogram was conducted. Axial reconstructions were performed with a slice thickness of 1.5 mm.

Two radiologists with six years of clinical experience each reviewed all CT chest images. The images were reviewed independently, and any discrepancy was resolved by consensus. There was no major disagreement between radiologists' interpretations.

For each of the two group patients, the CT exams were evaluated for the following characteristics: (1) CT severity scores (SS) for Initial and follow-up CT as described by Li et al. [8]; (2) ground-glass opacities and consolidations; and (3) presence of fibrotic lesions (traction bronchiectasis, parenchymal bands, honeycombing, and reticulations).

The CT SS is based on a visual quantitative evaluation of the percentage of involvement in each lobe and the overall lung. The SSs classified as none (0%), minimal (1%-25%), mild (26%-50%), moderate (51%-75%), or severe (76%-100%), with the corresponded score as 0, 1, 2, 3, or 4. The SS was reached by summing the five lobe scores (range from 0 to 20).

In the “case group,” the CT performed during acute illness is termed “the initial CT” and CT performed after the 10th week from the first PCR is termed “the follow-up CT.” On the other hand, follow-up CT image was not obtained in most patients in the “control group” as they showed significant clinical improvement; thus, the first CT performed during acute illness is termed “the initial CT” and the latest CT subsequently performed (not necessarily after 10 weeks of the acute illness) is termed “the follow-up CT.”

 Definitions of clinical status

The disease is considered moderate if the patient developed COVID-19 pneumonia without any severe or critical criteria. Severe disease is defined as COVID-19 pneumonia with any of the following: respiratory rate is ≥30/min, blood oxygen saturation is ≤93%, arterial oxygen partial pressure in mmHg to fractional inspired oxygen (PaO_2_/FiO_2_) ratio is <300, or radiological lung infiltration more than 50% of the lung field as judged on a CT chest. Cases that had a respiratory failure that required non-invasive or invasive ventilation or had Sepsis or septic shock, altered consciousness, or multi-organ failure were considered critical.

Statistical analysis

Statistical analyses were performed using STATA/IC version v16.1 software (StataCorp LLC, TX, USA). For normally distributed variables, means and standard deviations (SDs) were used to describe continuous variables, and frequencies and percentages were used for categorical variables. Non-normally distributed variables were described using median and interquartile ranges. Comparisons across groups were made using chi-square or Fisher's exact test for categorical variables and one-way ANOVA or Kruskal-Wallis test as appropriate for continuous variables. Logistic regression was used to evaluate risk factors associated with post-COVID-19 parenchymal lung changes. Multivariate models were adjusted for age, sex, smoking history, body mass index >30, and asthma to eliminate possible confounding factors. The variables were selected based on previous studies and clinical relevance. Statistical significance was set at P < 0.05.

## Results

A total of 38 patients were included in the study, 19 patients were included in each group. Patients' demographic and clinical characteristics are shown in Table 1. Patients with persistent parenchymal lung changes (group 1) were predominantly males (73.68%), however, was not statically significant between both groups. Age, BMI, smoking history, and comorbidity matched with insignificant P-value.

**Table 1:** Baseline characteristics of controls and cases NLR: neutrophil-to-lymphocyte ratio; ESR: erythrocyte sedimentation rate; CRP: C-reactive protein; LDH: Lactate dehydrogenase; RDW: red cell distribution width.

	Control (Group-2) No. 19	Case (Group-1) No. 19	P-value
Demographic data and comorbidities			
Age (years) (SD)	54.36 (12.14)	59.74 (13.82)	0.33
Gender: Male (%)	10 (52.63)	14 (73.68)	0.18
Smoking History (%)	4 (21.05)	4 (21.05)	0.65
Body mass index >30 (%)	11 (57.89)	10 (52.63)	0.74
History of diabetes (%)	10 (52.63)	11 (57.89)	0.74
History of hypertension (%)	10 (52.63)	11 (57.89)	0.74
History of chronic heart disease (%)	5 (26.32)	5 (26.32)	0.99
History of renal impairment (%)	3 (15.79)	2 (10.53)	0.63
History of chronic obstructive pulmonary disease (%)	1 (5.26)	0 (0)	0.31
History of Asthma (%)	4 (21.05)	3 (15.79)	0.68
History of active malignancy (%)	1 (5.26)	3 (15.79)	0.29
Inflammatory Biomarker Characteristics			
ESR (SD)	69.48 (36.33)	82.21 (40.92)	0.24
CRP (IQ)	7.2 (4.9-12.7)	12.45 (5.92-16.85)	0.23
Procalcitonin (IQ)	0.15 (0.11-0.58)	0.27 (0.09-0.43)	0.78
D-dimer (IQ)	1.24 (0.63-2.36)	2.00 (0.78-7.88)	0.21
LDH (SD)	363 (301-565)	480 (348-666)	0.22
Ferritin (IQ)	535 (262-1345)	711 (263-1407)	0.84
Lymphopenia at admission (%)	8 (42.11)	8 (42.11)	0.10
Admission NLR >3.13(%)	7 (36.84)	15 (78.95)	0.01
High Trop I during hospitalization (%)	3 (16.67)	2 (10.53)	0.59
RDW > or equal to 14.1 (%)	12 (63.16)	14 (73.68)	0.49
RDW value (IQ)	14.7 (12.9-16.3)	15.5 (13.7-17.3)	0.14

Neutrophil to lymphocyte ratio above 3.13 at admission was more prevalent in (group 1) 78.95% than in the control group (group 2) 36.84% with a significant P-value of (0.01). Group 1 has a higher peak value of inflammatory biomarkers, including erythrocyte sedimentation rate (ESR); C-reactive protein (CRP), procalcitonin, lactate dehydrogenase (LDH), and ferritin. However, these have insignificant P-values; the same applies to the D-dimer peak admission value.

About 84.3% had severe to critical acute COVID-19 infection in the control group (group 2) compared with 100% in group 1 (P-value 0.10). The majority of patients in both groups required admission to the ICU, 52.63% in group 2 and 68.42% in group 1. The number of ICU days, pharmacological treatment, and oxygen therapy other than mechanical ventilation are also comparable and statistically insignificant. Mechanical ventilation was strongly associated with persistent parenchymal lung changes; other hospital interventions and clinic-related clinical data are shown in Table 2.

**Table 2:** Inpatient and outpatient management related clinical data * Median ICU: intensive care unit; BiPAP: Bilevel Positive Airway Pressure; HFOV: High-frequency oscillatory ventilation.

	Control No.19	Case No.19	P-value
Admission related clinical data			
ICU admission (%)	10 (52.63)	14/13 (68.42)	0.31
Disease severity (%)			
Moderate	3 (15.79)	0 (0.00)	0.10
Severe	9 (47.37)	7 (36.84)	0.99
Critical	7 (36.84)	12 (63.16)	0.28
Received medications			
Interferon (%)	5 (26.32)	5 (26.32)	0.99
Lopinavir/ritonavir (%)	3 (15.79)	4 (21.05)	0.68
Ribavirin (%)	1 (5.26)	1 (5.26)	0.99
Convalescent Plasma (%)	0 (0.00)	3 (15.79)	0.07
Systemic steroid (%)	13 (68.42)	14 (73.68)	0.72
Tocilizumab (%)	6 (31.58)	11 (57.89)	0.10
Ventilatory support			
Nasal cannula /Face mask (%)	13 (68.42)	17 (89.47)	0.11
High flow nasal cannula (%)	6 (31.58)	8 (42.11)	0.50
BiPAP (%)	3 (15.79)	4 (21.05)	0.67
Mechanical ventilator (%)	1 (5.26)	8 (42.11)	0.008
HFOV (%)	1 (5.26)	2 (10.53)	0.55
Discharge related clinical data			
Systemic steroid after discharge (%)	9 (47.37)	15 (78.95)	0.043
Oxygen at discharge (%)	4 (21.05)	7 (36.84)	0.28
Readmission within 4 weeks of discharge (%)	2 (11.11)	5 (26.32)	0.23
Weeks on systemic steroid (SD)	4.00 (4.77)	9.11 (8.43)	0.03
Weeks on home oxygen^*^ (IQ)	0.00 (0.00-4.00)	0.00 (0.00-16.00)	0.19

The initial CT SS was higher in group 1 than the control group (13.06 and 9.58, respectively) (P = 0.043). However, after adjusting for age in the univariate and multivariate analysis, they were no longer significant with p-values of 0.05 and 0.08, respectively. Ground-glass opacities were detected in all cases, followed by parenchymal bands as the second most prevalent abnormality in nearly 80% of cases. None of the cases had a honeycomb appearance on chest CT imaging.

**Figure 1:**
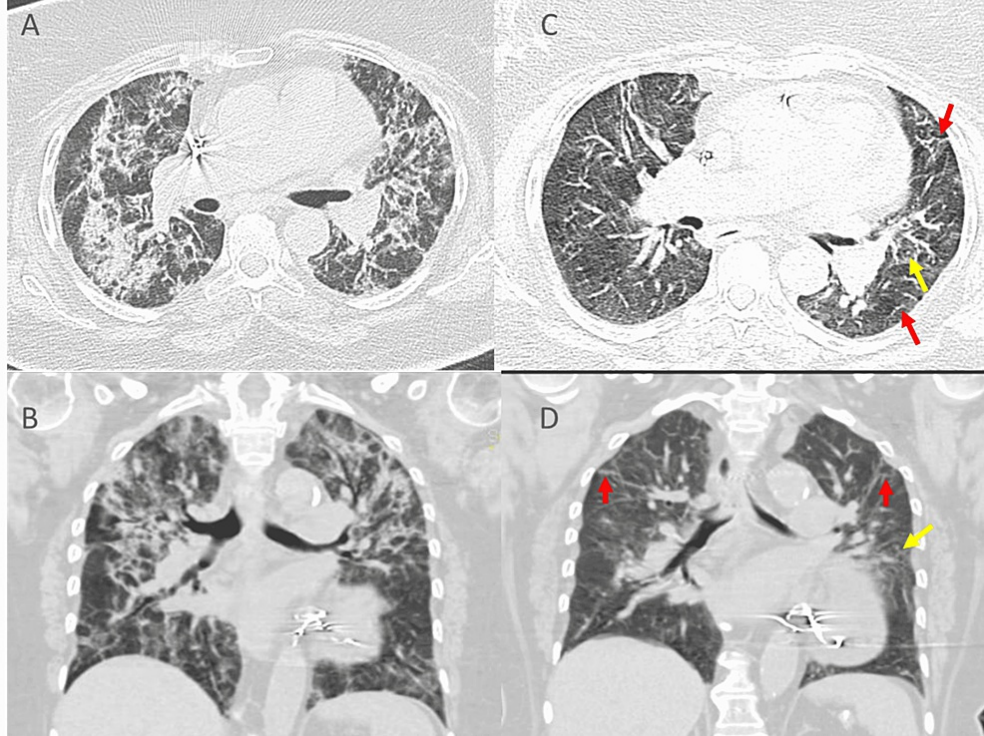
Representative CT scan of severe COVID19 infection in "group 1" (A, B) Initial CT chest, while (C, D) The follow-up CT (after 10 weeks) showing significant improvement, however, residual minimal patchy ground-glass opacities (yellow arrows), thickening of the interlobular septa, and linear opacities (red arrows) are noted in the bilateral lung.

**Figure 2:**
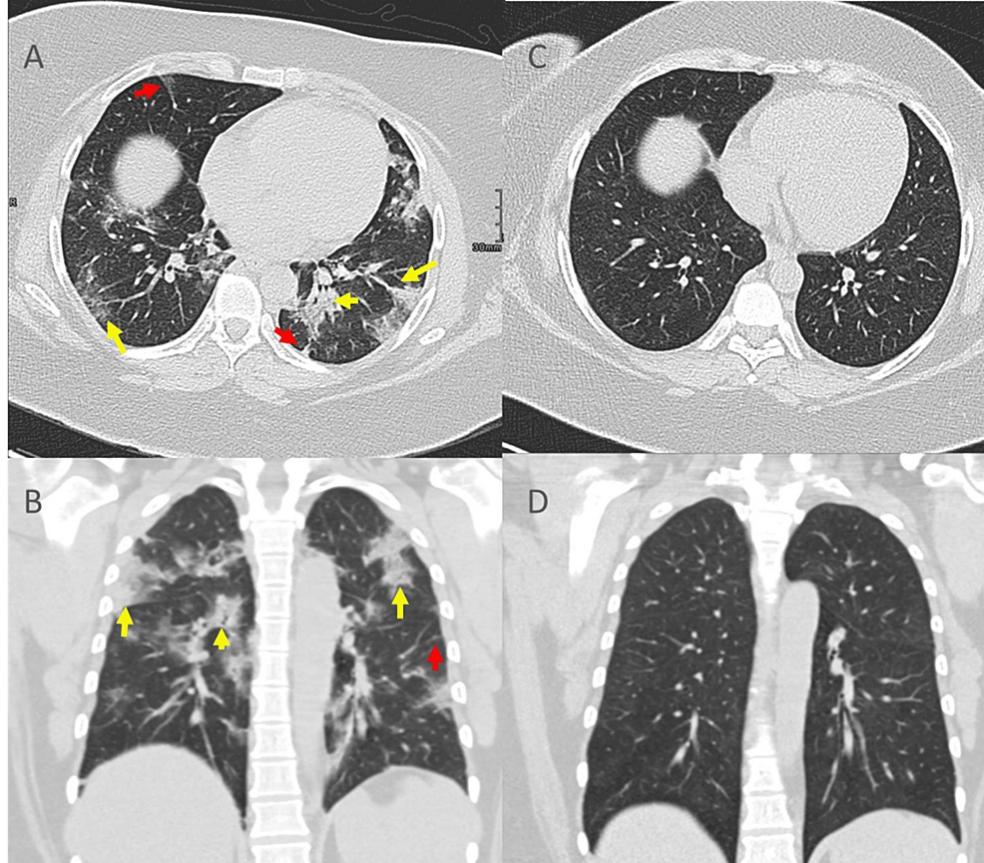
Representative CT scan of moderate COVID19 infection in "control group" (A, B) Initial CT chest shows patchy ground-glass opacities and focal consolidations (yellow arrows), with thickened interlobular septa (red arrows), in the bilateral lung. (C, D) Lesions were resolved in the follow-up CT.

The radiological SS and other persistent parenchymal abnormalities on follow-up chest CT are shown in Table 3 and illustrated in Figures 1A-1D, 2A-2D).

**Table 3:** Initial CT SS as a risk factor for post-COVID-19 fibrosis CT SS: CT severity score.

	Control (No.17)	Cases (No. 18)	P-value	Univariate OR	P-value	Multivariate OR	P-value
Initial CT SS (SD)	9.58 (4.17)	13.06 (5.45)	0.043	1.16 (0.99-1.34)	0.05	1.45 (0.98-1.34)	0.08
Adjusting for age (best-fitted model)							
Imaging characteristics of cases (Group-1) on the follow-up CT chest							
Follow up CT imaging characteristics			Case (Group-1) N=19				
Follow up CT performed Weeks (SD)			14.474 (4.55)				
Initial CT severity score (SD)			13.06 (5.45)				
Follow up CT severity score (SD)			9.84 (5.75)				
Traction bronchiectasis (%)			11 (57.89)				
Parenchymal bands (%)			15 (78.95)				
Honeycombing (%)			0.00 (0.00)				
Reticulations (%)			10 (52.63)				
Ground glass (%)			19 (100)				
Consolidation (%)			3 (15.79)				

The univariate and multivariate analyses of both groups for the possible risk factors of post-COVID-19 persistent parenchymal lung changes. Our analysis showed that a neutrophil-to-lymphocyte ratio (NLR) of > 3.13 and receiving invasive mechanical ventilation increased the odds ratio (OR) of persistent parenchymal changes. The OR were 6.42 and 13.09, respectively, in the univariate analysis and 5.92 and 44.5, respectively, in the multivariate analysis. Male sex, obesity, and comorbidity had a modest increase OR but were statistically insignificant. Other inflammatory markers and treatments are presented in Table 4.

**Table 4:** The univariate and multivariate analyses of both groups for the possible risk factors of post-COVID-19 persistent parenchymal lung changes Adjusting for age, gender, smoking history, BMI>30, Asthma ICU: intensive care unit; BiPAP: Bilevel Positive Airway Pressure; HFOV: High-frequency oscillatory ventilation; NLR: neutrophil-to-lymphocyte ratio; ESR: erythrocyte sedimentation rate; CRP: C-reactive protein; LDH: Lactate dehydrogenase.

	Univariate OR	P-value	Multivariate OR	P-value
Age (years)	1.034 (0.981-1.090)	0.210	1.048 (0.981-1.119)	0.167
Gender (male)	2.520 (0.646-9.833)	0.183	3.44 (0.72-16.48)	0.123
Smoking History	1.000 (0.210-4.758)	0.999	0.84 (0.13-5.67)	0.859
Body mass index >30	0.808 (0.225-2.909)	0.744	1.36 (0.29-6.36)	0.697
History of diabetes	1.24 (0.29-5.36)	0.500	0.82 (0.18-3.83)	0.804
History of hypertension	1.237 (0.344-4.454)	0.744	1.052 (0.213-5.195)	0.951
History of chronic heart disease	1.000 (0.236-4.238)	0.999	0.935 (0.160-5.459)	0.940
History of renal impairment	0.627 (0.092-4.259)	0.633	0.559 (0.053-5.898)	0.630
History of Asthma	0.703 (0.134-3.677)	0.677	0.92 (0.13-6.60)	0.942
History of active malignancy	3.38 (0.24-186.91)	0.302	1.93 (0.11-32.85)	0.216
Lymphopenia at admission	2.979 (0.789-11.248)	0.107	2.719 (0.617-11.982)	0.186
Admission NLR >3.13	6.429 (1.517-27.244)	0.012	5.928 (1.243-28.273)	0.026
High Trop I during hospitalization	0.588 (0.086-4.009)	0.588	0.311 (0.037-2.604)	0.282
ICU admission	1.950 (0.520-7.312)	0.322	1.232 (0.217-7.009)	0.814
PCR positivity for more than 14 days	0.791 (0.206-3.032)	0.733	0.686 (0.141-3.328)	0.640
Hydroxychloroquine	1.000 (0.175-5.720)	0.999	0.653 (0.093-4.566)	0.667
Favipiravir	2.187 (0.516-9.271)	0.288	2.509 (0.521-12.069)	0.251
Interferon	1.000 (0.236-4.238)	0.999	0.884 (0.186-4.208)	0.877
Lopinavir/ritonavir	1.422 (0.272-7.438)	0.677	0.902 (0.143-5.669)	0.912
Ribavirin	1.000 (0.058-17.249)	0.999	1.125 (0.046-27.285)	0.942
Systemic steroid	1.292 (0.317-5.275)	0.721	1.399 (0.289-6.757)	0.676
Tocilizumab	2.979 (0.789-11.248)	0.107	2.648 (0.593-11.834)	0.202
Nasal cannula /Face mask	3.923 (0.678-22.705)	0.127	2.914 (0.346-24.543)	0.325
High flow nasal cannula	1.576 (0.417-5.950)	0.502	1.148 (0.201-6.559)	0.877
BiPAP	1.422 (0.272-7.438)	0.677	1.129 (0.190-6.709)	0.894
Mechanical ventilator	13.091 (1.436-119.338)	0.023	44.542 (2.498-794.378)	0.010
HFOV	2.118 (0.176-25.549)	0.555	2.708 (0.148-49.436)	0.501
Respiratory bacterial infection	4.800 (0.483-47.682)	0.181	4.453 (0.377-52.610)	0.236
Other Systemic infection	1.000 (0.210-4.758)	0.999	0.877 (0.166-4.630)	0.877
Systemic steroid after discharge	4.167 (1.003-17.305)	0.049	3.034 (0.651-14.132)	0.157
Oxygen supplementation	2.187 (0.516-9.271)	0.288	1.627 (0.331-7.990)	0.550
Readmission within 4 weeks of discharge	2.857 (0.477-17.110)	0.250	1.778 (0.211-14.960)	0.597
ESR	1.009 (0.992-1.027)	0.316	1.005 (0.987-1.024)	0.573
CRP	1.061 (0.962-1.169)	0.237	1.061 (0.944-1.191)	0.320
Procalcitonin	0.767 (0.384-1.532)	0.452	0.833 (0.444-1.560)	0.567
D-dimer	1.063 (0.950-1.190)	0.286	1.063 (0.945-1.195)	0.309
LDH	1.002 (0.999-1.005)	0.216	1.002 (0.998-1.006)	0.280
Ferritin	1.000 (0.999-1.001)	0.774	1.000 (0.999-1.001)	0.418
Number of ICU days	1.062 (0.989-1.141)	0.098	1.067 (0.983-1.159)	0.122
Weeks on systemic steroid	1.121 (1.007-1.248)	0.038	1.126 (1.001-1.267)	0.048
Weeks on home oxygen	1.231 (0.936-1.618)	0.137	1.214 (0.925-1.595)	0.163

## Discussion

Persistent parenchymal lung changes after an acute COVID-19 infection are one of the most important post-COVID-19 sequelae. The risk factors leading to such a complication have not been well studied. Following previous outbreaks of coronaviruses, long-term pulmonary sequelae have been reported [9,10]. In a 12- month follow-up study of 311 patients with the severe acute respiratory syndrome (SARS), 21.5% of patients had lung fibrosis 65 days after discharge [11]. Das et al. reported that 13 out of 36 patients with Middle East respiratory syndrome (MERS) had persistent parenchymal changes 32-230 days after being discharged [12]. Chronic sequelae such as persistent symptoms, impaired diffusion capacity of the lung for carbon monoxide (DLCO), and persistent radiological changes all have been reported post-COVID-19 infection [13]. Trinkmann et al. reported that 113 out of 246 patients remained symptomatic after a mean follow-up period of 68 days; dyspnea was the most common symptom (32%) [14]. In Wuhan, a study showed impairment of DLCO after 90 days of discharge in 54% of patients [15]. In Norway, a multi-center prospective study reported that one-fourth of the patients had persistent CT findings on follow-up three months after discharge from acute COVID -19 hospitalization, ICU admission was reported to be a risk factor [16].

In this study, we studied 38 patients who were flowed at the ILD clinic for more than 10 weeks after COVID-19 infection. We have compared patients who had persistent parenchymal lung changes (group 1) with patients who had earlier clinical and radiological resolution (control group). Only one patient in the control group had a follow-up CT chest as per the study protocol (i.e., after 10 weeks from the positive PCR swab). This patient performed the scan 68 weeks after the acute illness, and it showed no abnormality other than a focal parenchymal scar. Another four patients in the control group had a follow-up CT, which was performed earlier than the 10th week. The rest of the control group had chest x-rays for follow up which did not suggest any chronic changes. Nineteen were confirmed by chest CT to have prolonged parenchymal abnormalities, i.e., parenchymal changes that lasted after at least 10 weeks of the acute infection and delayed clinical improvement.

The exact pathogenesis behind the development of lung fibrosis in COVID-19 survivors is not clear. It is likely that SARS-CoV-2 binds and interacts with an angiotensin-converting enzyme (ACE)-2, which increases transforming growth factor-beta (TGF β 1) and connective tissue growth factor (CTGF) levels, which may result in the development of fibrosis through the activation of fibrosis-related genes [17]. Surfactant abnormality and alveolar type-2 (AT2) cell injury resulting from the interaction between environmental factors, such as viruses and genetic factors, causing alveolar collapse, and repeated injury from ventilation could explain the progression to ventilator-induced lung injury (VILI) and lung fibrosis [18]. Evidence has shown that the most persistent ILD post-COVID-19 is reported in severe and critical cases [16,19-21]. This raises the suspicion that VILI and fibrosis following mechanical ventilation in acute severe respiratory distress syndrome have a major impact on the pathogenesis of post-COVID-19 ILD [18,22]. Our study is consistent with this observation, it showed that receiving invasive mechanical ventilation had the highest impact on the likelihood of developing prolonged parenchymal changes. It increases the odds ratio (OR) of chronic parenchymal changes by 13.09 and 44.5 in the univariate and multivariate analyses, respectively. Regarding disease severity, admission to the ICU and length of stay in the intensive care were not found to impact parenchymal lung sequelae; none of the medications administered for acute illness had an impact. However, CT-SS on initial chest CT was found to be higher in cases than in the control group, reflecting a more radiologically severe disease in group-1. After adjusting for age, the OR was found to be 1.16 (0.99-1.34), P = 0.05 and 1.45 (0.98-1.34), P = 0.08 in the univariate and the multivariate analysis, respectively.

An NLR of ≥ 3.13 was found to be an independent risk factor for severe and critical COVID-19 infection [23]. In this study, NLR was demonstrated to be a strong predictor of persistent parenchymal lung changes regardless of COVID-19 disease severity. There may be a role for increased neutrophils or decreased lymphocytes in disease pathogenesis and this requires further investigation. Moreover, a Swiss COVID-19 lung study evaluated pulmonary functions at four months of discharge and found that impaired DLCO was associated with previous severe to critical disease [19].

In patients with idiopathic pulmonary fibrosis (IPF), a study has shown that a red cell distribution width (RDW) of more than 14.1 was shown to be a negative prognostic factor as it correlates with lower forced vital capacity (FVC) and DLCO compared with IPF patients with normal RDW [24]. Increased RDW can be used as an indirect marker of hypoxemia. We did not find a difference in the percentage of patients who had RDW values above 14.1 between the groups. The absolute RDW value was also not different. We selected the value measured at the date of hospital discharge to allow time for acute illness-related hypoxemia to impact the RDW value.

In other coronavirus infections specifically MERS-CoV, post-infectious fibrotic lung changes were observed in patients that were admitted for longer days in the ICU, elderly patients, and those with higher LDH levels [12]. In contrast to what was observed in MERS-CoV, our study found that days of ICU admission, older age, and LDH level did not correlate with post-COVID-19 persistent parenchymal lung changes. Moreover, we found that prolonged viral shedding beyond 14 days had no direct impact on the development of persistent parenchymal lung changes after acute COVID-19 infection.

Limitations

It is essential to acknowledge the limitations of our study, which include retrospective design and the limited number of patients. We did not do CT chest for all control cases; however, clinical resolution of symptoms mirrors radiological changes resolution based on previous studies.

## Conclusions

An increasing number of long-term sequelae of the COVID-19 infection is being recognized over the past few months. Post-infectious ILD and chronic parenchymal lung changes are of particular importance as it has been reported to complicate pulmonary infection of similar viruses. We aimed to identify risk factors that increase the likelihood of developing persistent parenchymal lung changes. In this study, we have found that only receiving invasive mechanical ventilation, and NLR >3.13 at admission are strong risks for persistent parenchymal lung changes. Neither clinical severity of the acute illness nor the radiological one is found to predict this COVID-19-related complication. None of the medications received during the acute illness were found to alter the risk for this post-COVID-19 infection sequelae.
